# Wild and domestic animals variably display Neu5Ac and Neu5Gc sialic acids

**DOI:** 10.1093/glycob/cwac033

**Published:** 2022-06-01

**Authors:** Nikoloz Nemanichvili, Cindy M Spruit, Alinda J Berends, Andrea Gröne, Jolianne M Rijks, Monique H Verheije, Robert P de Vries

**Affiliations:** Division of Pathology, Department of Biomolecular Health Sciences, Faculty of Veterinary Medicine, Utrecht University, Yalelaan 1, 3584 CL Utrecht, The Netherlands; Department of Chemical Biology and Drug Discovery, Utrecht Institute for Pharmaceutical Sciences, Utrecht University, Universiteitsweg 99, 3584 CG Utrecht, The Netherlands; Division of Pathology, Department of Biomolecular Health Sciences, Faculty of Veterinary Medicine, Utrecht University, Yalelaan 1, 3584 CL Utrecht, The Netherlands; Division of Pathology, Department of Biomolecular Health Sciences, Faculty of Veterinary Medicine, Utrecht University, Yalelaan 1, 3584 CL Utrecht, The Netherlands; Dutch Wildlife Health Centre, Department of Biomolecular Health Sciences, Faculty of Veterinary Medicine, Utrecht University, Yalelaan 1, 3584 CL Utrecht, The Netherlands; Division of Pathology, Department of Biomolecular Health Sciences, Faculty of Veterinary Medicine, Utrecht University, Yalelaan 1, 3584 CL Utrecht, The Netherlands; Department of Chemical Biology and Drug Discovery, Utrecht Institute for Pharmaceutical Sciences, Utrecht University, Universiteitsweg 99, 3584 CG Utrecht, The Netherlands

**Keywords:** host tropism, influenza A, sialic acid, tissue microarray, wildlife

## Abstract

Sialic acids are used as a receptor by several viruses and variations in the linkage type or C-5 modifications affect the binding properties. A species barrier for multiple viruses is present due to α2,3- or α2,6-linked sialic acids. The C-5 position of the sialic acid can be modified to form *N*-acetylneuraminic acid (Neu5Ac) or *N*-glycolylneuraminic acid (Neu5Gc), which acts as a determinant for host susceptibility for pathogens such as influenza A virus, rotavirus, and transmissible gastroenteritis coronavirus. Neu5Gc is present in most mammals such as pigs and horses but is absent in humans, ferrets, and dogs. However, little is known about C-5 content in wildlife species or how many C-5 modified sialic acids are present on *N*-linked glycans or glycolipids. Using our previously developed tissue microarray system, we investigated how 2 different lectins specific for Neu5Gc can result in varying detection levels of Neu5Gc glycans. We used these lectins to map Neu5Gc content in wild Suidae, Cervidae, tigers, and European hedgehogs. We show that Neu5Gc content is highly variable among different species. Furthermore, the removal of *N*-linked glycans reduces the binding of both Neu5Gc lectins while retention of glycolipids by omitting methanol treatment of tissues increases lectin binding. These findings highlight the importance of using multiple Neu5Gc lectins as the rich variety in which Neu5Gc is displayed can hardly be detected by a single lectin.

## Introduction

It has been widely accepted that for many pathogens the crucial determinant in host species susceptibility is the sialic acid type and its linkage on target tissues. Sialic acids are variably present in tissues and secretions of different species and can contain a plethora of additional modifications (Matrosovich et al. 2000; Tumpey et al. 2007; Paulson and de Vries 2013; de Graaf and Fouchier 2014). These modifications influence host-related functions and many viruses, in turn, have adapted to specific sialic acid modifications (Rogers et al. 1986; Varki and Gagneux 2012; Song et al. 2016; Barnard et al. 2019; Broszeit et al. 2019; Tortorici et al. 2019; Morniroli et al. 2020).

One such modification is at the C-5 carbon, which can contain an *N*-acetyl or *N*-glycolyl structure (Varki et al. 2015), forming *N*-acetylneuraminic acid (Neu5Ac) and *N*-glycolylneuraminic acid (Neu5Gc), respectively . In mammals, the Neu5Gc modification is created via hydroxylation of Neu5Ac by the cytidine monophosphate-*N*-acetylneuraminic acid hydroxylase (CMAH) enzyme (Ito et al. 2000). This enzyme is expressed through the *CMAH* gene, which is present in many mammalian species but is non-functional in certain species such as dogs, ferrets, seals, hedgehogs, over a 100 different monkey species, and humans (Suzuki 2006; Ng et al. 2014; Springer et al. 2014; Peri et al. 2018). Furthermore, *CMAH* is also dysfunctional in white-tailed deer and 2 different bat lineages, 2 species that are important for severe acute respiratory syndrome coronavirus 2 (SARS-CoV-2) zoonosis (Chandler et al. 2021; Alkhovsky et al.  2022), which may be an interesting detail since sialic acids are suggested to be used as attachment mediating co-receptors by SARS-CoV-2 (Sun 2021; Nguyen et al. 2022). In contrast, mammals such as horses and pigs have a functional *CMAH* gene and display high levels of Neu5Gc (Suzuki et al. 1997, 2000). Previous research has shown that avian, human, and equine influenza A virus hemagglutinins (HAs) bind in an exclusive manner to either Neu5Ac or Neu5Gc (Connor et al. 1994; Broszeit et al. 2019; Spruit et al. 2022). Specificity is also seen for other pathogens such as rotavirus and transmissible gastroenteritis virus, where strains alter between Neu5Gc or Neu5Ac preference based on their host species (Schultze et al. 1996; Yu et al. 2012; Burzynska et al. 2021). Therefore, it is important to elucidate the biochemical display of Neu5Gc and Neu5Ac on the epithelial surface of domestic and wild animal tissues as glycobiology appears to play a key role in driving host-pathogen interactions.

We aimed to determine the Neu5Gc content on epithelial cells in wildlife with 2 different lectins, which are defined as glycoproteins capable of binding glycan structures found on glycoproteins and glycolipids. Therefore, both the antibody and recombinant viral attachment protein used in this study will be classified as lectins (Taylor et al. 2015). The anti-Neu5Gc IgY antibody (αNeu5Gc) was used previously and is available commercially (Paul et al. 2020), and an H5/Vietnam/1203/04 influenza A HA protein specific for Neu5Gc (H5VN^Y161A^ HA protein), which we produced and used frequently in previous studies (de Vries et al. 2014; Broszeit et al. 2019; Spruit et al. 2021) has been used as well. We determined the dependency of these 2 lectins on the glycan classes such as *N*-linked glycans or glycolipids. We then determined the Neu5Ac and Neu5Gc content on the respiratory tissues of wild animals including Suidae and Cervidae species, tiger (*Panthera tigris*), and European hedgehog (*Erinaceus europaeus*) and showed how 2 different lectins specific for Neu5Gc have varying specificities in animal tissues depending on the glycan classes.

Using lectin histochemistry, we observed that the αNeu5Gc and H5VN^Y161A^ HA protein can differentiate between the glycan classes. Wild Suidae has fluctuating levels of Neu5Gc and Neu5Ac content between the upper and lower respiratory tissues. In Cervidae, the receptor recognition of the αNeu5Gc antibody and H5VN^Y161A^ HA protein differed, with αNeu5Gc recognizing a Neu5Gc glycan that is not bound by H5VN^Y161A^ HA, highlighting why it is essential to use several lectins to analyze glycan contents on tissues. Overall, we show that Neu5Gc display is highly variable in the respiratory system of different species.

## Results

### The αNeu5Gc antibody displays batch variability in Neu5Gc binding

**Fig. 1:**
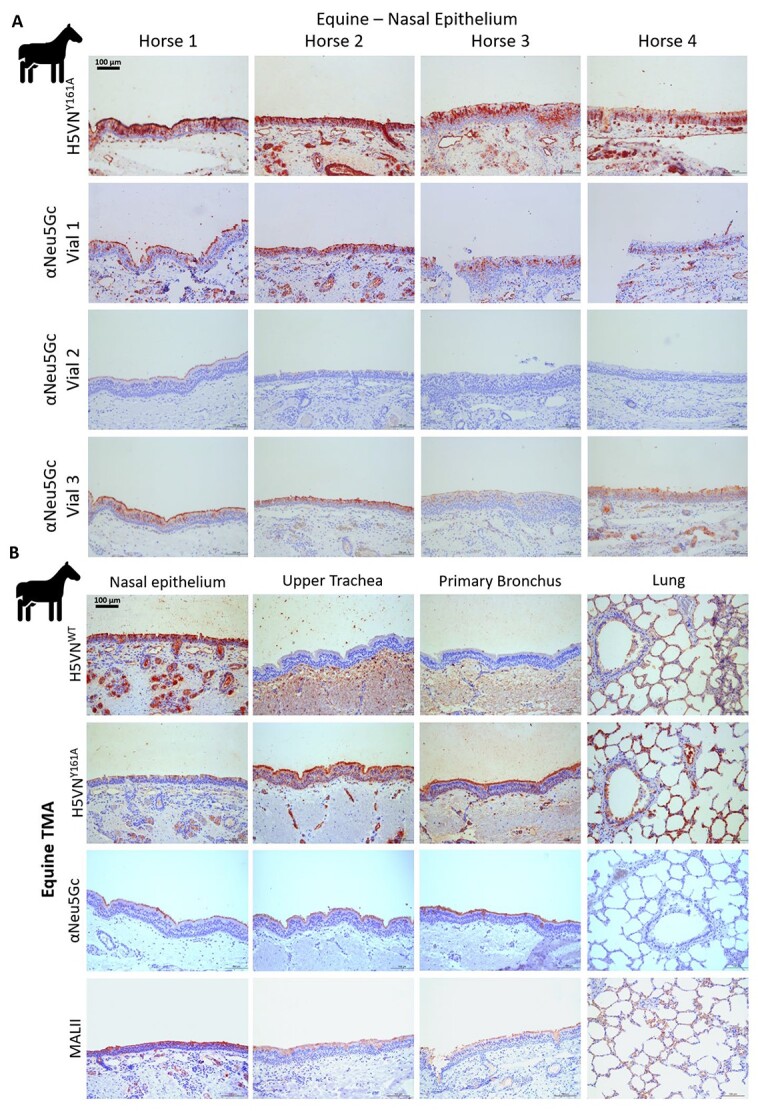
Comparison of αNeu5Gc antibodies and Neu5Gc & Neu5Ac specific HA proteins. **A**) Lectin histochemical staining of various equine nasal tissues with H5VN^Y161A^ and various batches of the αNeu5Gc IgY antibody to showcase batch variation in Neu5Gc detection. **B**) Staining of equine respiratory tissues with the H5VN^WT^ for α2,3-linked Neu5Ac presence, H5VN^Y161A^ & αNeu5Gc IgY (vial 3) for Neu5Gc presence and *Maackia amurensis* (MALII) lectin as a control for sialic acid presence.

We started by comparing the 2 Neu5Gc specific lectins, αNeu5Gc antibody and H5VN^Y161A^ HA protein, on the nasal tissue of horse, an established positive control animal for Neu5Gc detection (Broszeit et al. 2019; Spruit et al. 2022). The results showed that there was a difference in the binding consistency of the αNeu5Gc antibody between different vials (Fig. 1A). Histochemical staining with various αNeu5Gc antibody batches and H5VN^Y161A^ HA protein indicated that, while the H5VN^Y161A^ HA showed consistent binding over all nasal tissues in the horse tissue microarray (TMA), the αNeu5Gc antibody displayed varying binding properties, with vial 1 and 3 showing roughly similar binding patterns, whereas vial 2 shows no binding beyond the first horse (Fig. 1A). Histochemical stains with αNeu5Gc (vial 3) and H5VN^Y161A^ over the entire respiratory tract of the horse showed that the αNeu5Gc, while able to detect Neu5Gc presence in the epithelial layers, produced a much lower signal compared with H5VN^Y161A^, whereas the H5VN^WT^ HA shows that Neu5Ac is also present in the nasal epithelia of horse (Fig. 1B). These results show that the polyclonal nature of the αNeu5Gc antibody can be subject to vial variation. The αNeu5Gc antibody may also detect a narrower range of Neu5Gc receptors than the H5VN^Y161A^ stained the tissues deeper in the epithelial layer into the goblet cells in submucosal glands, suggesting there is Neu5Gc presence in goblet cells that the αNeu5Gc does not recognize. The difference in Neu5Gc range between the 2 lectins prompted us to analyze whether these lectins have a variable specificity to the different glycan classes.

### Removal of *N*-linked Neu5Gc glycans reduces binding of both the αNeu5Gc antibody and the H5VN^Y161A^ HA protein on equine respiratory tissues

**Fig. 2:**
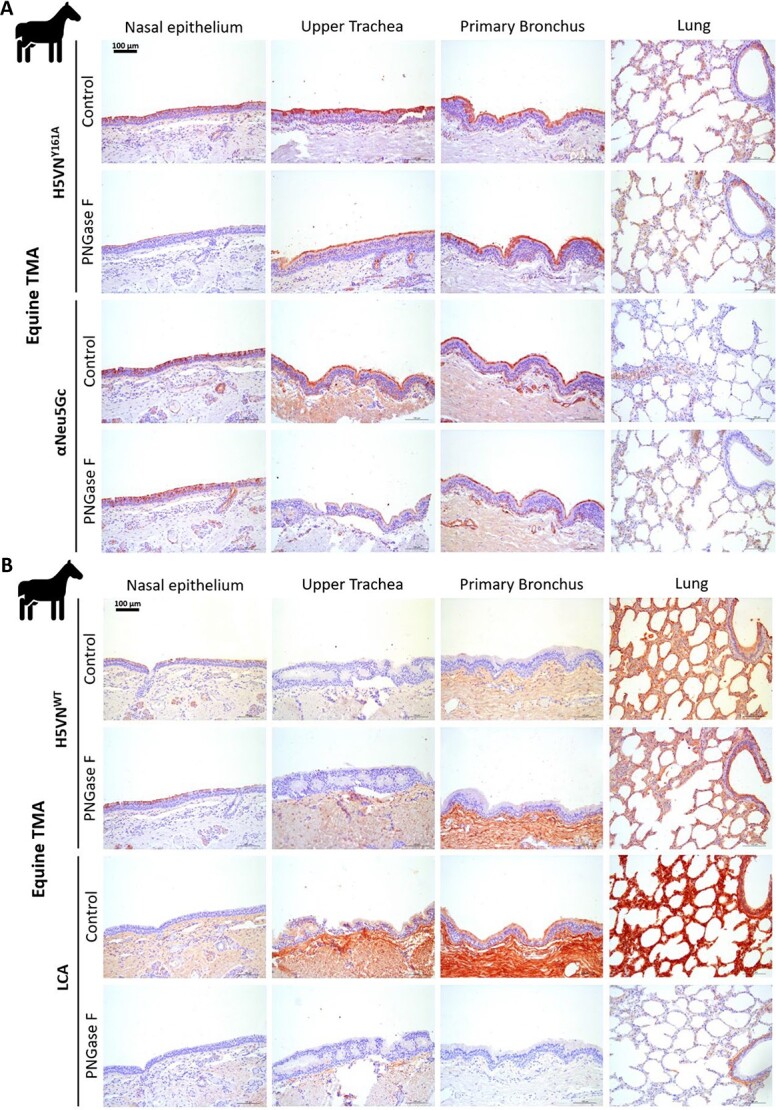
Lectin histochemistry of PNGase F treated tissues to remove *N*-linked glycans in equine. **A**) Lectin histochemical staining of H5VN^Y161A^ and αNeu5Gc IgY on PNGase F treated and untreated equine respiratory tissues to visualize dependency on *N*-linked glycans. **B**) Staining of PNGase F treated equine respiratory tissues with H5VN^WT^ for α2,3-linked Neu5Ac presence and *Lens culinaris* agglutinin (LCA) lectin as a control for the presence of *N*-linked glycans.

To test whether Neu5Gc is presented on *N*-glycans, we removed these *N*-glycans with PNGase F. Any residual binding by the lectins indicated *O*-linked glycans or glycolipid structures containing Neu5Gc. For both αNeu5Gc and H5VN^Y161A^, a slight reduction in binding affinity can be observed after PNGase F treatment of the horse respiratory tissues (Fig. 2A), however, neither of the Neu5Gc specific lectins lost binding completely, to any of the tissues. Two controls were performed to confirm these findings. The H5VN^WT^ HA (Fig. 2B), which is α2,3-linked Neu5Ac specific, showed that removal of *N*-linked glycans did not affect the presence of Neu5Ac sialic acids. The LCA (*Lens culinaris* agglutinin) lectin (Fig. 2B) specific for *N*-linked glycans showed that PNGase F did almost completely remove *N*-linked glycans. This confirms that although both αNeu5Gc and H5VN^Y161A^ can bind Neu5Gc containing *N*-linked glycans, the *N*-linkage is not an absolute requirement. Furthermore, the results suggest that a vast majority of binding comes from other glycan classes, potentially *O*-linked glycans or glycolipids. Indeed, the αNeu5Gc antibody was raised against a glycolipid (Diaz et al. 2009), whereas the H5VN^Y161A^ was serendipitously discovered after an alanine screen of conserved amino acid residues (Wang et al. 2012).

### Retention of glycolipids by omitting methanol treatment increases the binding of the H5VN^Y161A^ HA protein to equine respiratory tissues

**Fig. 3:**
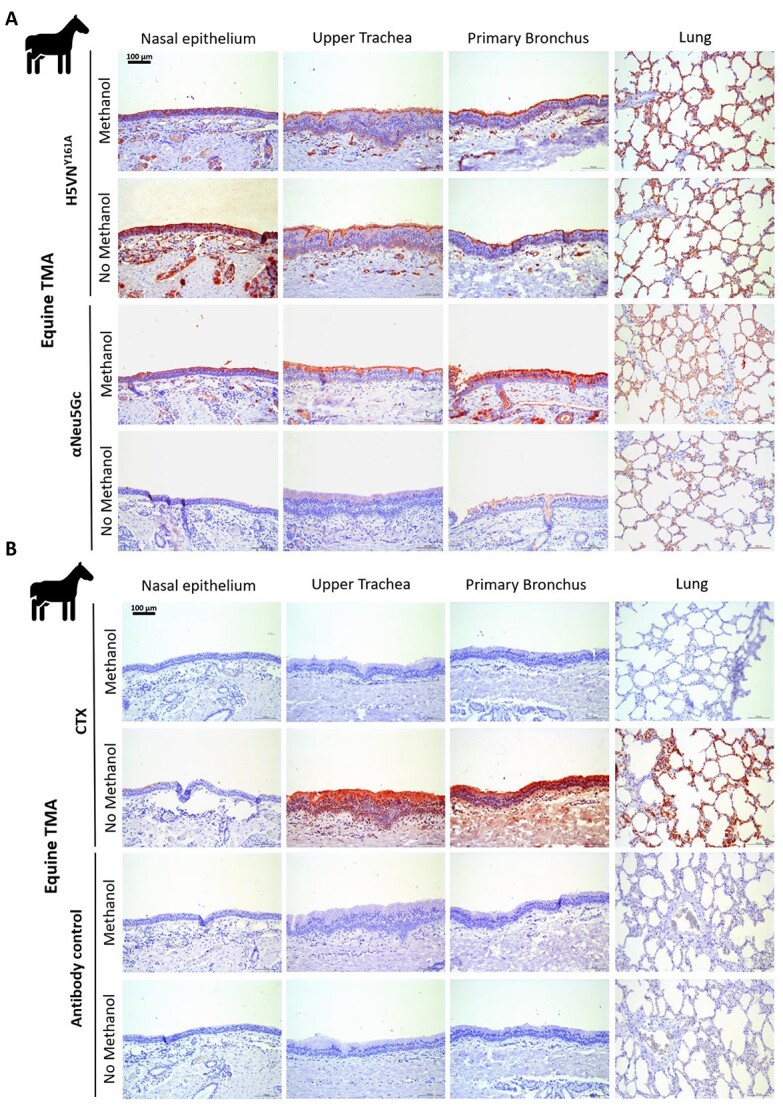
Lectin histochemistry of methanol-treated and untreated equine tissues to retain glycolipids. **A**) Lectin histochemical staining of H5VN^Y161A^ and αNeu5Gc IgY proteins on methanol untreated equine respiratory tissues to visualize dependency of glycolipids. **B**) Staining of methanol untreated equine respiratory tissues with Cholera Toxin subunit B lectin (CTX) as a glycolipid control and a precomplexed antibody-only control of α-strep-tag mouse-HRP and goat-α-mouse-HRP.

It is a common practice in tissue histochemical assays to treat tissues with methanol to remove any potential innate peroxidase activity (Bussolati and Radulescu 2011; Magaki et al. 2019). However, these treatments, along with the paraffin-embedding procedure, potentially remove glycolipids that are embedded in the epithelial membrane (Table 1). We, therefore, conducted histochemistry experiments in which we did not treat horse respiratory tissue with methanol to compare whether retention of glycolipids affected the binding properties of αNeu5Gc and H5VN^Y161A^ to Neu5Gc containing glycans. A comparison of H5VN^Y161A^ binding on methanol-treated and untreated tissue shows that untreated tissue gave a higher binding signal compared with methanol-treated tissues (Fig. 3A). Striking however is that untreated tissue gave a lower signal for the αNeu5Gc lectin (Fig. 3A). Cholera toxin subunit B (CTX) protein, specific for glycolipids, showed that treatment with methanol removes all glycolipids on the cell surface consistently and that there are almost no glycolipids present in nasal tissue (Fig. 3B). Meanwhile, the precomplexed antibody control without any HA protein confirmed that there is no nonspecific binding of antibodies to glycolipids. These results show that glycolipid binding by H5VN^Y161A^ is possible but not an absolute requirement for binding to horse respiratory tissues. Now with an established protocol that no longer uses methanol to detect Neu5Gc structures, we aimed to study its presence in understudied wildlife species.

**Table 1:** Tissue preparation, embedding, and processing steps with indications of potential loss of glycolipids and mucins.

**Process**	**Potential loss of glycolipids**	**Potential loss of mucins**
**Tissue fixation**		
Paraformaldehyde	✔	✔
Ethanol 70%		
**Dehydration & embedding**		
Ethanol 70%		
Ethanol 96%		
Xylene	✔	✔
Paraffin		
**Rehydration sectioned tissue**		
Xylene	✔	✔
Ethanol 96%		
Ethanol 70%		
Water		
**Histochemical staining treatments**		
Methanol treatment	✔	

### Both Suidae and Cervidae show a variance in Neu5Gc display

**Fig. 4:**
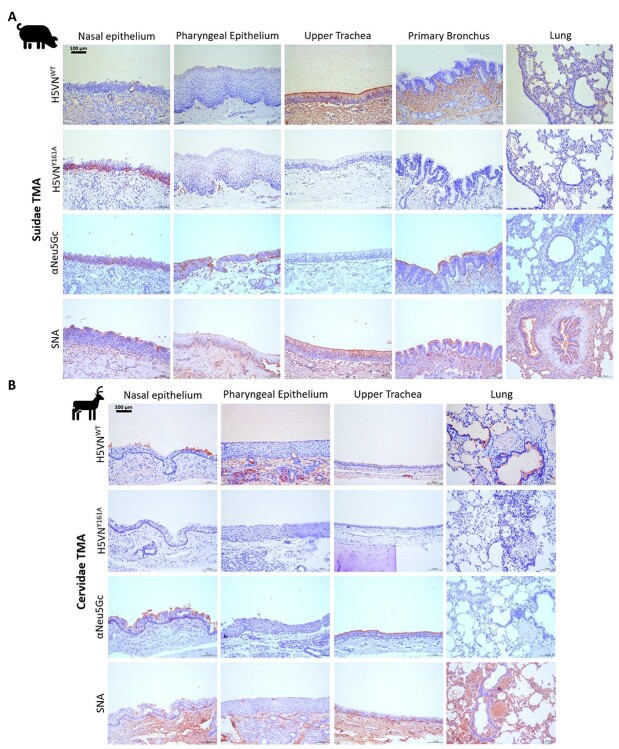
Lectin histochemistry for Neu5Ac and Neu5Gc detection on wild Suidae and Cervidae. **A**) Lectin histochemical staining of Suidae (*Sus scrofa* shown) respiratory tissues with H5VN^WT^ for α2,3-linked Neu5Ac presence, H5VN^Y161A^ & αNeu5Gc IgY for Neu5Gc presence and *Sambucus nigra* (SNA) lectin as a control for α2,6-linked sialic acid presence. **B**) Staining of Cervidae (*Capreolus capreolus* shown) respiratory tissues with the lectins indicated in A.

We then moved on to testing the Neu5Gc content in wild animals that can come into proximity to domestic species. For this, wild Suidae and Cervidae species were selected as their Neu5Gc content has not been mapped. Lectin histochemical stains showed that for wild Suidae (Fig. 4A, *Sus scrofa* shown) the content of Neu5Gc varied over the respiratory tract. Higher Neu5Gc concentrations were present in the nasal epithelium than further down the respiratory tract. The α2,6-linked specific SNA lectin showed binding over the entire respiratory tract. On the other hand the α2,3-linked Neu5Ac specific H5VN^WT^ protein gains binding lower into the respiratory tract. This suggests that there is a gradient of Neu5Gc to Neu5Ac in the respiratory tract of wild Suidae. For the Cervidae however a consistent lack of binding could be seen for H5VN^Y161A^ (Fig. 4B, *Capreolus capreolus* shown) as well as only sporadic binding of the α2,6-linked specific SNA lectin, but curiously the αNeu5Gc did display binding over the entire respiratory tract except for the lung. This indicates that some Neu5Gc containing structures are present in Cervidae, which are recognized by αNeu5Gc, but not by H5VN^Y161A^ HA. Lastly, the H5VN^WT^ did show binding on the Cervidae respiratory tract confirming that there is also α2,3-linked Neu5Ac present on the tissues in the epithelial layer alongside the Neu5Gc structures that the αNeu5Gc bound.

**Fig. 5:**
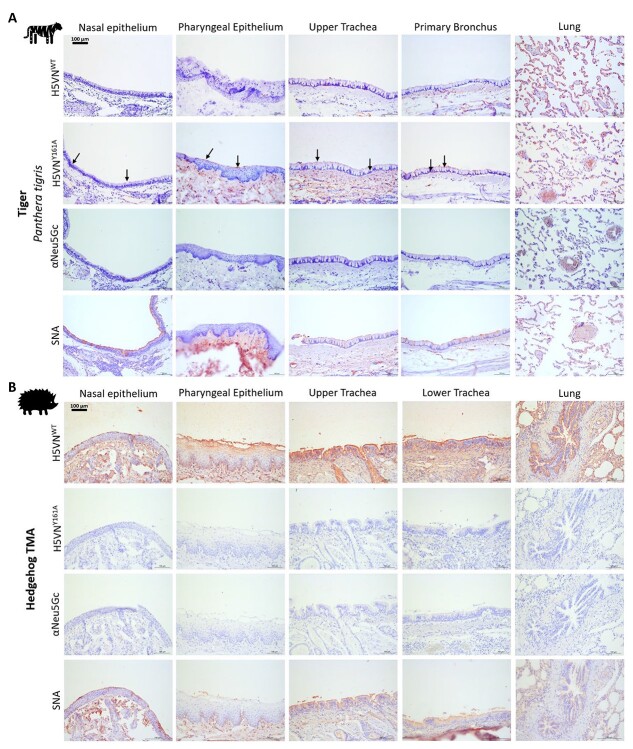
Lectin histochemistry for Neu5Ac and Neu5Gc detection on CMAH active and deficient species. **A**) Tiger respiratory tissues with H5VN^WT^ for α2,3-linked Neu5Ac presence, H5VN^Y161A^ & αNeu5Gc IgY for Neu5Gc presence and *Sambucus nigra* (SNA) lectin as a control for α2,6-linked sialic acid presence. The black arrows highlight locations of binding for H5VN^Y161A^. **B**) Staining of European hedgehog respiratory tissues with the lectins indicated in A.

### Tiger is a Neu5Gc species while European hedgehog is a Neu5Ac species

Lastly, 2 wildlife species were tested from which in literature the presence of an intact *CMAH* gene was studied. The tiger was marked as a species containing Neu5Gc possibly over the entire respiratory system, whereas the European hedgehog was expected to not contain any Neu5Gc (Peri et al. 2018). For the tiger, only the Neu5Gc specific H5VN^Y161A^ mutant HA and α2,6-linked specific SNA lectin showed binding over the entire respiratory tract (Fig. 5A), whereas in the European hedgehog (Fig. 5B) only the Neu5Ac specific H5VN^WT^ HA and α2,6-linked specific SNA lectin showed binding. These results in terms of Neu5Gc contents matched expectations found in the literature (Table 2; Peri et al. 2018). However, while H5VN^Y161A^ did show binding in tiger respiratory tissues αNeu5Gc did not, demonstrating again that there are Neu5Gc structures that only 1 of the 2 Neu5Gc lectins recognizes.

### Evaluation of the presence of intact *CMAH* genes

For the formation of Neu5Gc, an intact CMAH enzyme is required, but the presence of a seemingly intact *CMAH* gene does not automatically lead to (high) expression of the CMAH enzyme, and therefore presence of Neu5Gc (Jahan et al. 2021). Previously, the presence of intact *CMAH* genes was investigated for species of which a reference genome is available (Peri et al. 2018). It was shown that horses, wild boar, roe deer, and tigers have intact *CMAH* genes, whereas there is an incomplete *CMAH* gene in European hedgehogs. For some species (peccary and red river hog), no genome is available and thus the presence of an intact *CMAH* gene is unclear. We investigated whether intact *CMAH* genes were present in the other animals investigated in this article (Table 2), of which the genomes were present in the whole-genome shotgun database (Supplementary Table S1), which are the red deer, hog deer, and reindeer. In the red deer and hog deer, complete *CMAH* genes were observed. In reindeer, one exon could not be found and therefore we cannot be certain whether the *CMAH* gene yields an active enzyme. However histochemical staining results for reindeer matched the results found in Cervidae (Fig. 4A and B) suggesting that the complete *CMAH* gene is present and does yield active enzymes.

## Discussion

In this study, we used a Neu5Gc specific HA protein of H5VN and an αNeu5Gc antibody to explore the Neu5Gc content in the respiratory tract of domestic and wild animals. Lectin histochemistry on horse respiratory tissue showed that the H5VN^Y161A^ and αNeu5Gc antibody can bind both *N*-linked glycans and glycolipids containing Neu5Gc, however, signals from the HA proteins were more intense. Importantly, we found a considerable vial variation between the αNeu5Gc antibodies. We, therefore, as with many glycan determinants, recommend using multiple lectins for a similar target.

Testing of wild animals such as wild Suidae and Cervidae shows that, just as with domesticated animals such as horses, wild animals can contain both Neu5Ac and Neu5Gc (Suzuki et al. 1985; Barnard et al. 2020). With the wild Suidae, the display of Neu5Gc and Neu5Ac changes when descending from the upper respiratory tract into the lower parts, seeing a gradual switch from Neu5Gc to Neu5Ac. This contrasts with what is seen in domestic Suidae which are entirely Neu5Gc, save for the lungs (Suzuki et al. 1997; Burlak et al. 2013; Byrd-Leotis et al. 2014). These results highlight the differences between domesticated and wild species and how pathogen susceptibility might therefore vary between the 2. Interestingly, in Cervidae we observed the first difference between the 2 Neu5Gc lectins with H5VN^Y161A^ displaying no binding while the αNeu5Gc did. This suggests that the CMAH enzyme in Cervidae does not convert a specific subset of Neu5Ac to Neu5Gc, one that is not recognized by the HA protein. Although it remains unknown which specific Neu5Gc structures are present on the epithelial surface, these findings do again highlight why it is essential to use several different lectins to analyze Neu5Gc glycan display on tissues.

The results found with the methanol treatments also show the importance of taking glycolipids into account as they can contain Neu5Gc presenting structures. When it comes to glycolipids it is important to note that the process of creating paraffin-embedded tissues comes with a loss of secreted mucins and surface glycolipids (Table 1), 2 structures that can not only contain Neu5Gc but are also factors that play a key role in pathogen evasion and tropism via (decoy) receptors. For mucins, it is known that paraffin-embedding does not lead to a total loss as mucin histochemical stainings are still possible using Periodic acid–Schiff (PAS) and Alcian Blue stain methods (Ali et al. 2013). Loss of glycolipids is also a known occurrence from paraffin-embedding procedures (Alroy et al. 1986), however, to but is not complete as we found that some glycolipids are still present using cholera toxin when excluding the methanol treatment from histochemistry. To which extent this paraffin-embedding influences glycolipid Neu5Ac and Neu5Gc display on respiratory tissues is unknown as Neu5Gc glycolipid presence can vary per species, and is still poorly understood. Histology using frozen tissue methods however demonstrates that mucin and glycolipid retention is far superior when using cryo-embedded tissues methods embedding compared to paraffin-embedding (Cohen et al. 2012). The downside however is that cryo-embedding requires fresh tissue samples collected quickly after the animal has died, which is not always possible when working with exotic animals and was beyond the possible scope of this study.

Lastly, in tiger and European hedgehog, we see that tiger is a Neu5Gc species, whereas European hedgehog is a Neu5Ac species. These findings show that wildlife species can also have an entire respiratory tract containing only Neu5Gc or Neu5Ac in terms of receptor presentation. Curiously for tiger, we see again a difference between the 2 Neu5Gc lectins with the H5VN^Y161A^ HA protein being able to bind, whereas the αNeu5Gc antibody cannot. This is most likely caused by a subclass of Neu5Gc glycans that the polyclonal αNeu5Gc does not recognize. This could be tied to the blood group as Neu5Gc is a blood group determinant in domesticated cats (Andrews et al. 1992; Bighignoli et al. 2007). Finally, we demonstrate that European hedgehogs do not contain Neu5Gc as suspected due to an incomplete *CMAH* gene.

Overall, we show that Neu5Gc content is highly variable in different species, highlighting the need of mapping the loss of *CMAH* functionalities in different mammals. Mapping these highly variable contents is important as it will shed light on potential pathogen evasion strategies and potential animals with a low species barrier for zoonosis. We also highlight the importance of using multiple Neu5Gc lectins for mapping as the rich variety in which Neu5Gc is displayed can lead to certain lectins being unable to bind a specific glycan class of Neu5Gc.

## Material and methods

### Expression vectors and protein production

Human codon-optimized HA sequences from A/Vietnam/1203/04 (H5N1) (GenBank: EF541403)—for both the H5VN^WT^ and H5VN^Y161A^ mutant—were cloned into the pCD5 vector as previously described (de Vries et al. 2010; Nemanichvili et al. 2021). Both the plasmids have been submitted to Addgene as pCD5-H5_Vietnam_1203/04-sfGFP_ST (H5VN^WT^, catalog #182546) and pCD5-H5_Vietnam_1203/04-Y161A-sfGFP_ST (H5VN^Y161A^, catalog #182547). The resulting pCD5-HA-GCN4-sfGFP-Strep encodes for the hemagglutinin of the HA protein in frame with a trimerization domain, superfolder green fluorescent protein (sfGFP), and Strep-tag located C-terminally. The H5VN^Y161A^ mutant was generated by site-directed mutagenesis (Q5, New England Biolabs, Ipswich, MA, United States) of the HA using primers introducing a single point mutation in the DNA codon changing tyrosine at position 161 into alanine (Y161A; Broszeit et al. 2019). Both HA proteins were produced using a mammalian cell culture system as previously described (de Vries et al. 2010; Nemanichvili et al. 2021).

**Table 2:** Overview *CMAH* gene status in all tested domestic and wild animal species from literature (Peri et al. 2018).

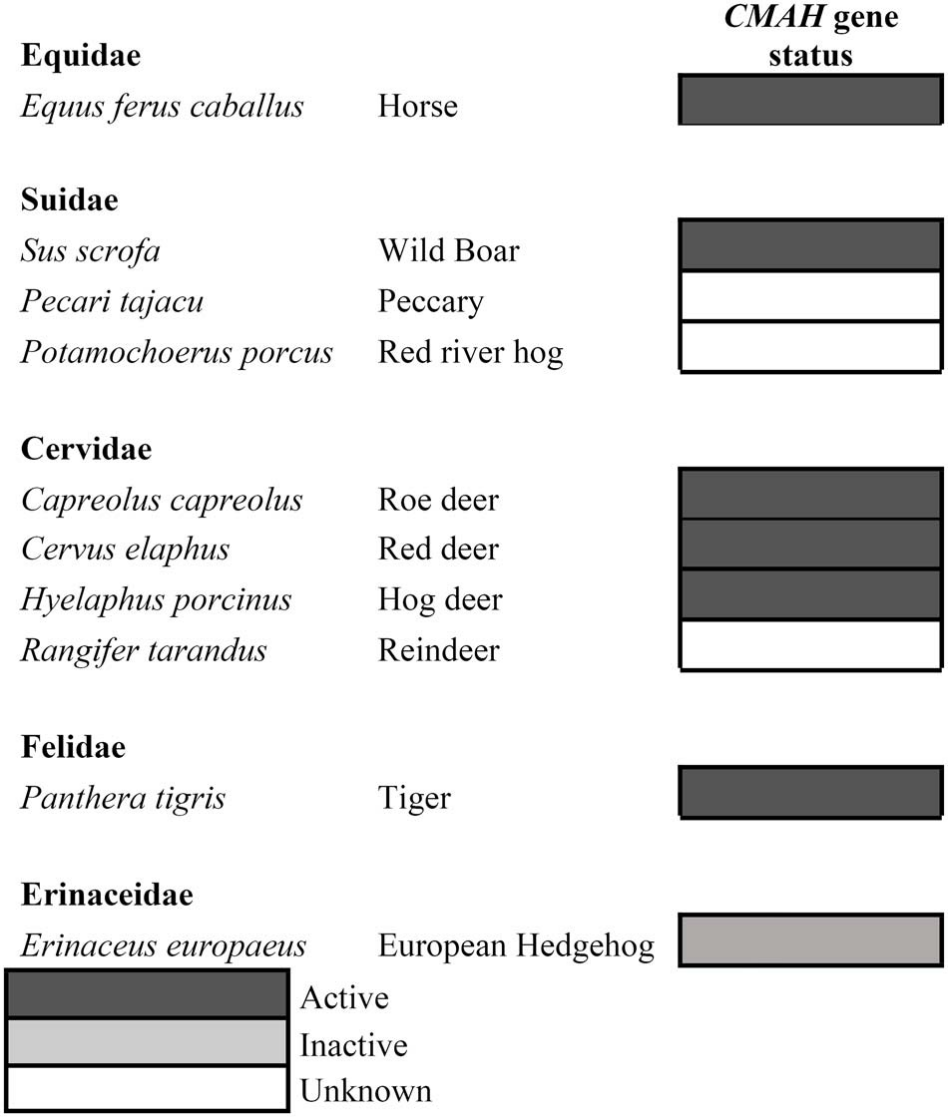

### Tissue collection and TMAs creation using 3D-printed array plates

Tissues originating from the respiratory tract (nasal epithelium, pharyngeal epithelium, upper trachea, lower trachea, primary bronchus, and lung) of selected species were collected from and assembled in 5 TMAs (Table 3). Tissues were obtained from dead animals, which were sent for diagnostic and educational purposes to the Veterinary Pathologic Diagnostic Center, Division of Pathology, Faculty of Veterinary Medicine, Utrecht University. No animals were killed for this study. After collection, tissues were fixed in buffered pH 6.9 formaldehyde solution 4% m/v for 24–48 h at room temperature and subsequently transferred into a 70% ethanol solution for storage. Tissues were processed according to standard paraffin-embedding procedures (Fischer et al. 2008), in brief, the fixed tissues were dehydrated in steps through 70% ethanol, 96% ethanol, and xylene followed by embedding into paraffin blocks, these procedures are known to likely remove some glycolipids and secreted mucins during the process, 2 factors that play key roles in viral tropism (Table I). Sections cut from these tissue blocks onto slides were then deparaffinized and rehydrated in steps through xylene, 96% alcohol, 70% alcohol, and water. Microscopic evaluation of hematoxylin and eosin-stained slides was performed to determine tissue quality before selection and punching of tissue cores for incorporation into microarrays using a 2-mm biopsy punch pen (Miltex). These tissue cores were then incorporated into an empty 3D-printed array plate.

**Table 3:** The species composition of the 5 tissue microarrays.

**TMA**	**English name**
**TMA 1 (Equine)**	
*(5x) Equus ferus caballus*	Horse
**TMA 2 (Suidae)**	
*(4x) Sus scrofa*	Wild boar
*(1x) Pecari tajacu*	Peccary
*(1x) Potamochoerus porcus*	Red river hog
**TMA 3 (Cervidae)**	
*(2x) Capreolus capreolus*	Roe deer
*(2x) Cervus elaphus*	Red deer
*(1x) Hyelaphus porcinus*	Hog deer
*(1x) Rangifer tarandus*	Reindeer
**TMA 4 (exotic wildlife)**	
*(1x) Panthera tigris*	Tiger
**TMA 5 (Erinaceidae)**	
*(5x) Erinaceus europaeus*	European hedgehog

The 3D-printed array plates were designed as previously described (Nemanichvili et al. 2021). Once the plate was filled with the required amount of tissue cores it was embedded into paraffin and left to cool. These TMA blocks were then cut into thin 0.4-μm slices onto microscopy slides for immunohistochemical staining.

### Lectin histochemical staining

Tissue slides were prepared for lectin histochemical staining as previously described (Wickramasinghe and Verheije 2015; Nemanichvili et al. 2018). Tissues were treated for 30 min in methanol with 1% v/v peroxidase to inhibit endogenous peroxidase activity. For tissue staining 5 μg/mL of the HA proteins were precomplexed with α-strep-tag mouse-horseradish peroxidase (HRP; IBA Lifesciences, Göttingen, Lower Saxony, Germany) primary antibody and goat-α-mouse-HRP (Thermofisher, Waltham, MA, United States) secondary antibody at 4:2:1 molar ratio as previously described (Nemanichvili et al. 2021). Hematoxylin stains were performed to visualize tissue structure and morphology before covering the stained slides with coverslips using AquaTex (Merck). For stains with αNeu5Gc, tissues were stained with a 1:300 dilution of αNeu5Gc IgY (Biolegend, San Diego, CA, United States) followed by 1:100 dilution of goat anti-chicken IgY-HRP (Thermofisher, Waltham, MA, United States). For initial testing 3 different vials of αNeu5Gc were used to test batch variance. Vial 1 was from an earlier batch while vials 2 and 3 were from the same batch. After initial testing shown in Fig. 1A, it was vial 3 that was used for subsequent stains. For removal of *N*-linked glycans, tissues were treated overnight with 50 μL/mL PNGase F (New England Biolabs, Ipswich, MA, United States) in a humidity chamber at 37°C before being stained. The lectin *L. culinaris* Agglutinin (LCA; Vector Laboratories, Burlingame, CA, United States) was used at 2 μg/mL as a control for *N*-linked glycan presence. For retention of glycolipids, the 30-min treatment of tissues in methanol with 1% v/v peroxidase was omitted. The recombinant HRP conjugated Cholera Toxin Subunit B (CTX) (Thermofisher, Waltham, MA, United States) of 1 μg/μL was used at a 1:200 dilution as a control for glycolipid presence. The lectins *Sambucus nigra* agglutinin (SNA) and *Maackia amurensis* (MALII; Vector Laboratories, Burlingame, CA, United States) were used at 2.5 and 5 μg/mL, respectively to confirm the presence of sialic acids on the tissue. All lectin histochemical stains and controls were performed 3 times in independent assays (*n* = 3).

### Analysis of the *CMAH* genes

The *CMAH* genes of the horse (*Equus caballus*), roe deer (*C. capreolus*), wild boar/domestic pig (*S. scrofa*), tiger (*P. tigris*), and European hedgehog (*E. europaeus*) were already investigated previously (Peri et al. 2018). No genome was available for the red river hog (*Potamochoerus porcus*) and peccary (*Pecari tajacu)*. We analyzed the *CMAH* genes of the red deer (*Cervus elaphus*), hog deer (*Axis porcinus*), and reindeer (*Rangifer tarandus*). The genomes were present in the whole-genome shotgun contigs (WGS) collection. Using blastn, we searched for the 15 exons of the chimpanzee (*Pan troglodytes*) *CMAH* gene in the respective genome, with a search-optimized for more dissimilar sequences (discontinuous megablast). The first exon was too small to find significant similarity and the 15th exon always gave >100 hits. Therefore, these 2 exons were not analyzed. The presence, orientation, and order of the exons was investigated (Supplementary Table S1 and Supplementary File S1). The exons were aligned with the chimpanzee exons and additional bases were added when the blastn search did not indicate the complete exon. Finally, the presence of stop codons in the exons was investigated. A stop codon was detected in all analyzed animals in exon 14 and therefore this was considered an artifact, possibly still yielding an active CMAH enzyme.

## Supplementary Material

Supplementary_file_S1_cwac033

Supplementary_table_SI_cwac033

## Abbreviations

CMAH, cytidine monophospho-*N*-acetylneuraminic acid hydroxylase; CTX, cholera toxin subunit B;, HA, hemagglutinin; HRP, horseradish peroxidase; H5VN, H5 HA from A/Vietnam/1203/2004; LCA, *Lens culinaris* agglutinin; MALII, *Maackia amurensis* lectin II; Neu5Ac, *N*-acetylneuraminic acid; Neu5Gc, *N*-glycolylneuraminic acid; sfGFP, superfolder green fluorescent protein; SNA, *Sambucus nigra* lectin; TMA, tissue microarray; WGS, whole-genome shotgun
